# The secreted protein acidic and rich in cysteine (SPARC) induces endoplasmic reticulum stress leading to autophagy-mediated apoptosis in neuroblastoma

**DOI:** 10.3892/ijo.2012.1678

**Published:** 2012-10-24

**Authors:** G.S. SAILAJA, PRAVEEN BHOOPATHI, BHARATHI GORANTLA, CHANDRAMU CHETTY, VENKATESWARA RAO GOGINENI, KIRAN KUMAR VELPULA, CHRISTOPHER S. GONDI, JASTI S. RAO

**Affiliations:** 1 Departments of Cancer Biology and Pharmacology, and; 2 Neurosurgery, University of Illinois College of Medicine at Peoria, Peoria, IL 61605, USA

**Keywords:** secreted protein acidic and rich in cysteine, neuroblastoma, autophagy, apoptosis, endoplasmic reticulum stress

## Abstract

Our previous studies showed that overexpression of secreted protein acidic and rich in cysteine (SPARC) induced autophagy-mediated apoptosis in PNET cells. In the present study, we attempted to elucidate the molecular mechanisms and signaling cascades associated with SPARC overexpression in combination with radiation therapy that eventually leads to autophagy-mediated apoptosis in neuroblastoma. SPARC expression in SK-N-AS and NB-1691 cells demonstrated the activation of caspase 3, cleavage of PARP and induction of apoptosis. The experiments to unravel the mechanisms associated with autophagy-apoptosis illustrated that SPARC overexpression triggered endoplasmic reticulum (ER) stress and thereby unfolded protein response (UPR). This was apparent with the activation of stress receptors, inositol-requiring enzyme (IRE 1α), RNA-dependent protein kinase (PKR)-like ER kinase (PERK) and BiP. This study further demonstrated the induction of transcription factor CHOP as a result of IRE-JNK activation in response to increased SPARC levels. Inhibition of ER stress and JNK activation led to inhibition of autophagy-mediated apoptosis. Further, the apparent expression of ER stress molecules among the orthotopic tumors treated by SPARC overexpression plasmids substantiated our *in vitro* observations. Taken together, these results illustrate the critical role of ER stress in regulating autophagy-mediated apoptosis in SPARC-overexpressed neuroblastoma cells and radiation treatment.

## Introduction

Neuroblastoma, the most common extracranial pediatric malignancy, is characterized by a broad spectrum of clinical behaviors with a low survival rate (1,2). The clinical prognosis reveals its aggressive behavior, including wide dissemination, resistance to chemotheraphy, and ability to metastasize to the bone (3). Since the tumor is associated with a high fatality rate, various investigators are working to improve the survival rate, reduce recurrence, and metastasis of neuroblastoma. Like any other malignant tumor, the hallmarks of high-risk neuroblastoma are unregulated cellular proliferation and declined apoptosis. Apoptosis is the intrinsically programmed cell death that is necessary to maintain normal homeostasis of the system. Its diminution violates many of the physiological checkpoints inside the cell and elicits abnormal behavior in the cellular microenvironment. The potentially detrimental effects observed with the absence/reduction of apoptosis can be considered as a function of the activation of anti-apoptotic signaling cascades (4). Given the important role of apoptosis regulators in neuroblastoma, a better understanding of the underlying mechanisms involved in the induction of apoptosis would be a very important aspect in designing anticancer drugs and developing efficient therapeutic strategies.

Secreted protein acidic and rich in cysteine (SPARC) is an evolutionary matricellular glycoprotein composed of three structural domains with distinct modular functions and plays critical roles in modulating cell-matrix interactions, cellular functions and tissue mineralization (5). SPARC plays a significant role in tissue remodeling, embryogenesis, cellular differentiation and angiogenesis. As a matricellular protein, SPARC takes part in multiple biological activities including development and regulation of matrix remodeling. As such, molecular mechanisms associated with variation in SPARC levels in the cell would have decisive consequences in regulating the diverse cellular functions.

The underlying mechanisms involved in SPARC expression and malignant tumor regression remain elusive; however, it is known that SPARC expression is highly dependent on various factors including tumorigenic phenotype, molecular signaling pathways associated with integrins and growth factors/chemokines (5). Even though the comprehensive role of SPARC-mediated tumor regression is not completely understood, previous studies have clarified the anti-proliferative and counter adhesive properties of SPARC through specific cytokines and growth factors (6–8).

The significance of combination therapy arises from the possibility of achieving a synergistic effect in advanced treatment modalities. Different combination therapies, like combination of chemotherapy and radiation therapy, have been investigated to improve the efficiency (9). Radiation has been recognized as an efficient mode of therapy for neuroblastoma and provides an additive pharmacological response when combined with other treatments (10,11).

Our recent findings showed SPARC overexpression inhibited cell proliferation, migration and angiogenesis in PNET cells (12,13). Earlier reports from our group also proved that SPARC overexpression induced autophagy-mediated apoptosis in PNET cells (14). However, the molecular mechanisms associated with SPARC overexpression leading to autophagy-mediated apoptosis have not been studied. In the present study, we attempted to elucidate the efficacy of SPARC overexpression together with radiation therapy as an efficient way to induce apoptosis in neuroblastoma as well as investigate associated molecular mechanisms.

This study provides solid evidence describing the key role of endoplasmic reticulum (ER) stress in invoking the transcriptional responses as a function of SPARC overexpression. ER is the sole element responsible for protein synthesis and folding; an imbalance between the cellular demand and the capacity of ER to facilitate protein folding eventually activates aberrant cell cycle regulation, deregulates the intercellular metabolism, and eventually activates ER stress molecular chaperons. Our study was also focused on understanding the specific pathways associated with SPARC overexpression that induce ER stress, which in turn elicit autophagy-mediated apoptosis in neuroblastoma. The induction of autophagy as a result of ER stress has been identified by various investigators (15,16). Autophagy involves sequestration of autophagosomes that eventually fuse with lysosomes and lead to cellular degradation; as a result, autophagy has an important role in eukaryotic cells. Hence, we also investigated the involvement of autophagy as an event associated with SPARC overexpression and induction of apoptosis in neuroblastoma.

## Materials and methods

### Cell culture

SK-N-AS and NB-1691 cells were procured from ATCC (Manassas, VA) and Dr P. Houghton of St. Jude Children’s Research Hospital (Memphis, TN), respectively. The cells were maintained in RPMI-1640 supplemented with 10% FBS (Invitrogen Corp., Carlsbad, CA), 50 U/ml penicillin and 50 *μ*g/ ml streptomycin (Life Technologies, Inc., Frederick, MD). Cells were maintained in a 37°C incubator with a 5% CO_2_ humidified atmosphere.

### Antibodies and reagents

The primary antibodies against SPARC, caspase 3, LC3, JNK, phospho-JNK, pancreatic ER kinase (PERK), GAPDH (Santa Cruz Biotechnology, Santa Cruz, CA), PARP, inositol-requiring enzyme 1α (IRE-1α), BiP, and CHOP (Cell Signaling Technology, Beverly, MA) were used in this study. HRP- or Alexa Fluor-488/Alexa Fluor-594-conjugated secondary antibodies, isotype control IgG (Santa Cruz Biotechnology, Santa Cruz, CA), Vectashield mounting medium with DAPI (Vector Laboratories, Burlingame, CA), DAB peroxidase substrate (Sigma, St. Louis, MO), TUNEL (terminal deoxynucleotidyl transferase-mediated dUTP nick-end-labeling) detection kit (Roche Molecular Biochemicals, Indianapolis, IN), JNK Inhibitor II SP600125 (Calbiochem, San Diego, CA), and Salubrinal (ER stress inhibitor, Fisher) were also used in this study.

### Construction of pcDNA3.1-SPARC, transfection and irradiation of neuroblastoma cells

Human SPARC cDNA was amplified by PCR using synthetic primers and was cloned into a pcDNA3.1 vector (Invitrogen, San Diego, CA) in sense orientation as described previously (17). Neuroblastoma cells were transfected with plasmid vector containing full-length cDNA of SPARC (pSPARC) or empty vector (pEV) using FuGene HD (Roche, Indianapolis, IN) as described earlier (18). After 4–6 h of transfection, the necessary amount of serum-containing medium was added. After 24 h of incubation, cells were irradiated with X-ray irradiation at a dose of 8 Gy using the RS 2000 Biological Irradiator (Rad Source Technologies, Inc., Boca Raton, FL). Then, the medium replaced, and cells were incubated for a further 16 h or for the indicated time period.

### Immunocytochemistry

Immunocytochemistry was performed as described previously (17). Briefly, cells were cultured on 8-well chamber slides and transfected as above. Forty-eight hours after transfection, cells were fixed with 4% freshly prepared paraformaldehyde (w/v) in PBS followed by permeabilization with 0.1% Triton X-100 (w/v) in PBS and blocked with 1% BSA (w/v) in PBS for 1 h at 4°C. Cells were incubated overnight at 4°C with anti-SPARC or anti-CHOP antibody followed by corresponding Alexa Fluor-488- or Alexa Fluor-594-conjugated secondary antibody for 1 h at room temperature. Slides were mounted with Vectashield mounting medium with DAPI (Vector Laboratories) and analyzed under a microscope (Olympus BX61 Fluoview, Minneapolis, MN). Isotype control IgG served as a negative control.

### Western blotting

Western blot analysis was performed as reported earlier (19). Briefly, 48 h after transfection, cells were collected and lysed in RIPA buffer. Equal amounts of protein were resolved on SDS-PAGE and transferred onto a PVDF membrane. The blots were blocked with 5% non-fat dry milk and probed overnight with primary antibodies followed by HRP-conjugated secondary antibodies. ECL system was used to detect chemiluminescent signals. All blots were re-probed with GAPDH antibody to confirm equal loading.

### Reverse transcription polymerase chain reaction (RT-PCR)

Neuroblastoma cells were transfected with mock, pEV or pSPARC for 36 h. Total RNA was extracted from these cells and cDNA synthesized using poly-dT primers as described earlier (20). PCR was performed using the following primers: SPARC: 5′-GGAAGAAACTGTGGCAGAGG-3′ (sense), and 5′-ATTGCTGCACACCTTCTCAA-3′ (antisense); GAPDH: 5′-TGAAGGTCGGAGTCAACGGATTTGGT-3′ (sense), and 5′-CATGTGGGCCATGAGGTCCACCAC-3′ (antisense).

### Flow cytometry

FACS analysis was used to assess cell cycle phases (FACSCalibur System, BD Bioscience, San Jose, CA) with laser excitation at 488 nm and an emission at 639 nm band pass filter to collect red propidium iodide fluorescence. The percentages of cells in the various phases of the cell cycle (sub-G0/G1, S, and G2/M) were assessed using Cell Quest software (BD Bioscience, San Jose, CA).

### Terminal deoxy nucleotidyl transferase-mediated nick labeling (TUNEL) assay and immunohistochemistry

Apoptosis in neuroblastoma cells after SPARC transfection alone and in combination was detected using TUNEL enzyme reagent according to the manufacturer’s instructions and as described previously (17). Briefly, 2×10^3^ cells were cultured in 4-well chamber slides, transfected with pSPARC, irradiated after 24 h of transfection, fixed in 4% paraformaldehyde in PBS for 1 h at room temperature, and permeabilized in 0.1% Triton X-100 in 0.1% sodium citrate in PBS for 10 min on ice. The samples were incubated in TUNEL reaction mixture in a humidified atmosphere at 37°C for 1 h in the dark. Slides were mounted and images were captured with an Olympus BX 60 research fluorescence microscope attached to a CCD camera, and cells were counted. The apoptotic index was defined as follows: apoptotic index (%) = 100 × (apoptotic cells/total cells).

### Intra-adrenal tumor model and immunohistochemistry

The Institutional Animal Care and Use Committee at the University of Illinois College of Medicine at Peoria approved all experimental procedures involving the use of animals. Orthotopic, localized neuroblastoma tumors were established in C.B-17 SCID mice by injection of 1×10^6^ NB-1691 cells in 100 *μ*l PBS into the retroperitoneal space as described earlier (21). After 2 weeks of tumor cell implantation, the mice were separated into six groups containing 6 animals per group, and each group was injected intravenously with PBS (mock), pEV or pSPARC (100 *μ*l volume) and were given three doses on alternate days. Between the first and the second injections, and the second and the third injections, one group was irradiated with a dose of 5 Gy each time. Mice were euthanized when they lost >20% of body weight or had trouble ambulating, feeding, or grooming. The tumors were removed and either fixed in 10% phosphate-buffered formaldehyde or snap frozen and maintained at −70°C until sectioning. Briefly, all tumors were serially sectioned and tissue sections (5-*μ*m thick) obtained from the paraffin blocks were stained with hematoxylin and eosin (H&E) using standard histologic techniques. For immunohistochemical analysis, sections were incubated with primary antibody for 2 h at room temperature followed by the HRP-conjugated secondary antibody. DAB solution was used as the chromogen. Isotype control IgG was used as a negative control. The nucleus was counterstained with hematoxylin and sections were mounted and analyzed.

### Statistical analysis

All data are presented as means ± standard error (SE) of at least three independent experiments, each performed at least in triplicate. One way analysis of variance (ANOVA) combined with the Tukey *post hoc* test of means was used for multiple comparisons in cell culture experiments. Statistical differences are presented at probability levels of p<0.05, p<0.01 and p<0.001.

## Results

### SPARC overexpression followed by radiation therapy enhanced apoptosis in neuroblastoma cells

**Figure 1.**
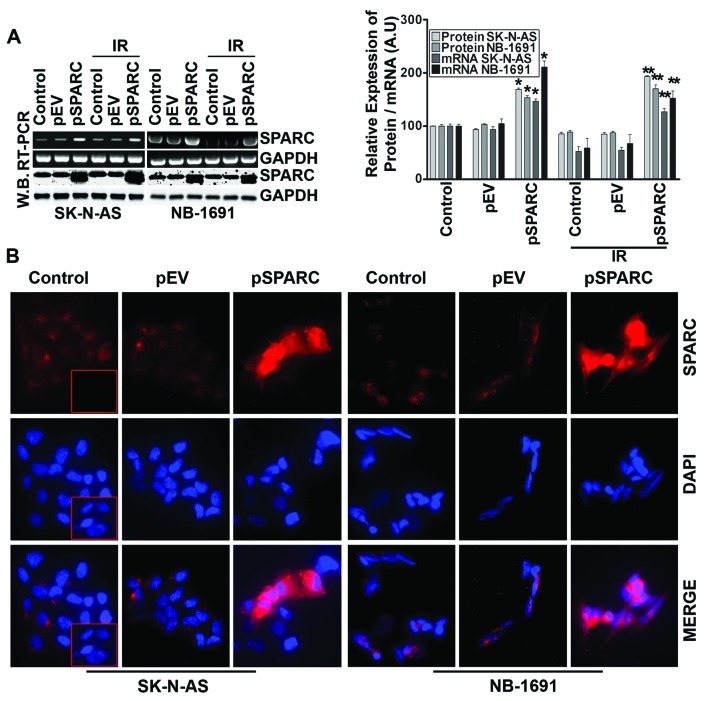
SPARC overexpression in neuroblastoma cells. (A) SK-N-AS and NB-1691 neuroblastoma cells were seeded in dishes and left overnight. Cells were transfected with pEV or pSPARC and cultured. After 24 h, cells were irradiated with 8 Gy and incubated for another 16 h. Total RNA was extracted from the control, transfected and irradiated cells. RT-PCR was performed for SPARC using specific primers. GAPDH served as a control. SPARC levels were determined by western blot analysis using a SPARC-specific antibody. GAPDH served as a loading control. Results are representative of three independent experiments. Densitometric analysis showing levels of SPARC protein and mRNA levels. Columns, mean of three experiments; bar, ± SD. ^*^p<0.01 vs. pEV; ^**^p<0.01 vs. IR + pEV. (B) Neuroblastoma cells were cultured in 8-well chamber slides and transfected with pEV or pSPARC. After 36 h, cells were fixed, blocked and incubated overnight with anti-SPARC primary antibody followed by incubation with Alexa Fluor-594-conjugated secondary antibody for 1 h. Slides were mounted with Vectashield mounting medium containing DAPI and photographed under a confocal microscope. Inset, negative control with isotype control IgG.

**Figure 2.**
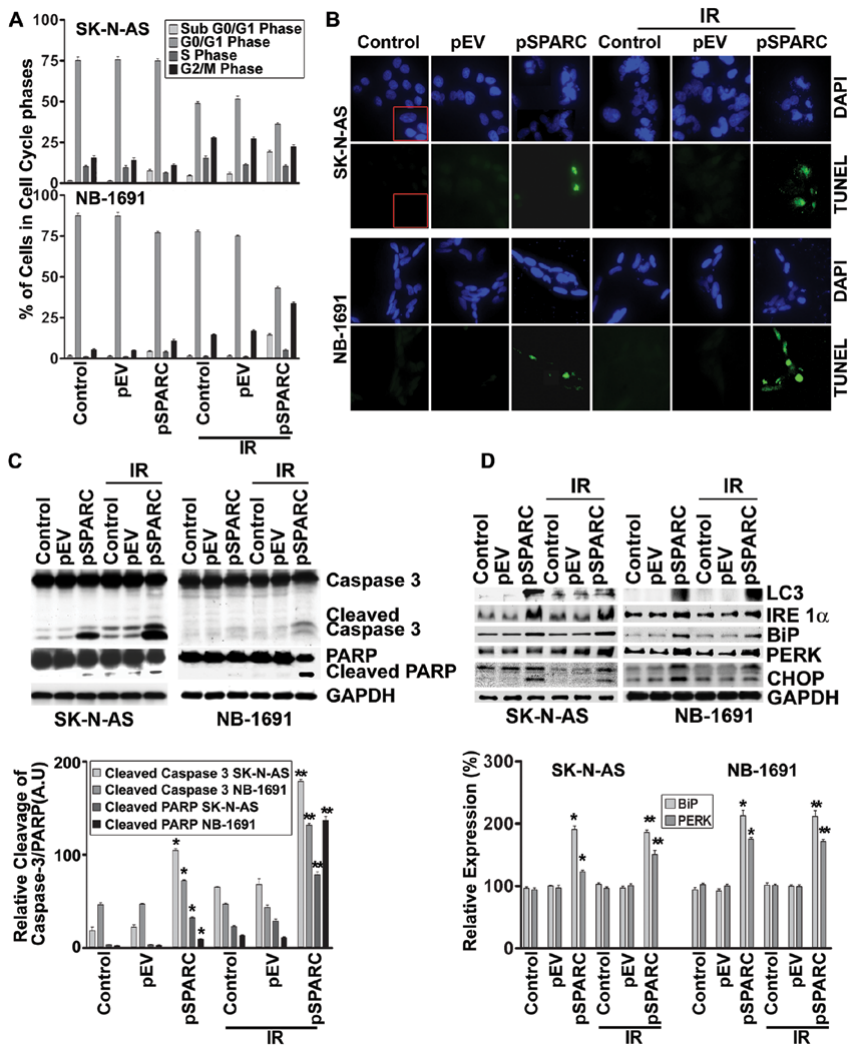
SPARC overexpression sensitizes cells to radiation in neuroblastoma cells. SK-N-AS and NB-1691 neuroblastoma cells were seeded in dishes and left overnight. Cells were transfected with pEV or pSPARC and cultured. After 24 h, cells were irradiated with 8 Gy and incubated for another 16 h. (A) Cells were collected and subjected to FACS analysis with propidium iodide staining for DNA content analysis and are represented in a graphical manner. Columns: mean of three experiments; bars, ± SD. ^*^p<0.01 vs. pEV; ^**^p<0.01 vs. IR + pEV. (B) Neuroblastoma cells were cultured in 8-well chamber slides, transfected and irradiated as described above. Cells were fixed with paraformaldehyde, permeabilized and blocked. Cells were incubated with TUNEL reaction mixture for 1 h. Nuclei were counterstained with DAPI and slides were mounted and photographed. Inset, negative control. (C) Western blot analysis was performed for caspase 3 and PARP from total cell lysates using specific antibodies. GAPDH served as a loading control. Densitometric analysis showing levels of cleaved caspase 3 and PARP. Columns, mean of three experiments; bars, ± SD. ^*^p<0.01 vs. pEV; ^**^p<0.01 vs. IR+pEV. (D) Total cell lysates were used for western blot analysis to detect protein levels for LC3, IRE 1α, BiP, PERK and CHOP using specific antibodies. GAPDH served as a loading control. Densitometric analysis showing levels of BiP and PERK. Columns: mean of three experiments; bars, ± SD. ^*^p<0.01 vs. pEV; ^**^p<0.01 vs. IR + pEV.

It has been demonstrated that SPARC overexpression induces apoptosis in PNET cells (14). In this study, initially, the role of SPARC overexpression to induce apoptosis by itself and in combination with radiation was investigated in neuroblastoma cells. The expression of SPARC significantly increased at both protein and mRNA levels in cells transfected with pSPARC (Fig. 1A). The expression was increased by >75% in both cell lines with and without radiation combination when compared to the respective control or empty vector-treated counterparts (Fig. 1A). SPARC expression levels after transfection were also checked by immunofluorescence analysis, which also demonstrated an apparent increase in cellular expression of SPARC in pSPARC-transfected cells (Fig. 1B). Further, flow cytometric analysis showed that SPARC transfection alone or in combination with radiation (IR) dosage of 8 Gy resulted in a significant increase of the sub-G0/G1 population of cells, which indicates the induction of apoptosis in the SK-N-AS and NB-1691 neuroblastoma cells (Fig. 2A). SPARC and IR-induced apoptosis was further confirmed by TUNEL assay (Fig. 2B) and cleavage of caspase 3 and PARP (Fig. 2C). These results demonstrate that SPARC overexpression increased the sensitivity of neuroblastoma cells to radiation.

### SPARC overexpression induces autophagy

Our earlier studies showed that SPARC overexpression led to autophagy-mediated apoptosis in PNET cells (14). To understand the molecular pathways associated with SPARC overexpression leading to autophagy, expression of autophagy marker protein microtubule-associated protein light chain-3 II (LC3-II), which is formed as a result of phosphoatidylethanolamine conjugation of LC3-I was chosen (22). Increased expression of LC3 was observed for SPARC-overexpressed neuroblastoma cell lines, which confirms autophagy as a part of the molecular events leading to apoptosis (Fig. 2D). To better understand the cellular pathways associated with SPARC overexpression leading to autophagy-mediated apoptosis, the role of endoplasmic reticulum (ER) stress (22) was investigated. IRE 1, a type 1 ER transmembrane bifunctional glycoprotein having serine/threonine kinase and endoribonuclease activities in its cytosolic domain, was found to be upregulated with SPARC overexpression (Fig. 2D). In addition, other ER molecular chaperons, BiP and PERK, were also found to be activated with increased SPARC expression at an early time period of 24 h in NB-1691 and SK-N-AS cell lines (Fig. 2D). The prolonged stress led to the upregulation of the proapoptotic transcription factor, CHOP, which was induced as a result of DNA damage and/or other stress conditions (Fig. 2D). A significant increase of these molecules in pSPARC treated cells indicated a direct involvement of autophagy in SPARC induced apoptosis in neuroblastoma.

### Endoplasmic reticulum stress activates the c-Jun N-terminal kinase (JNK) pathway

**Figure 3.**
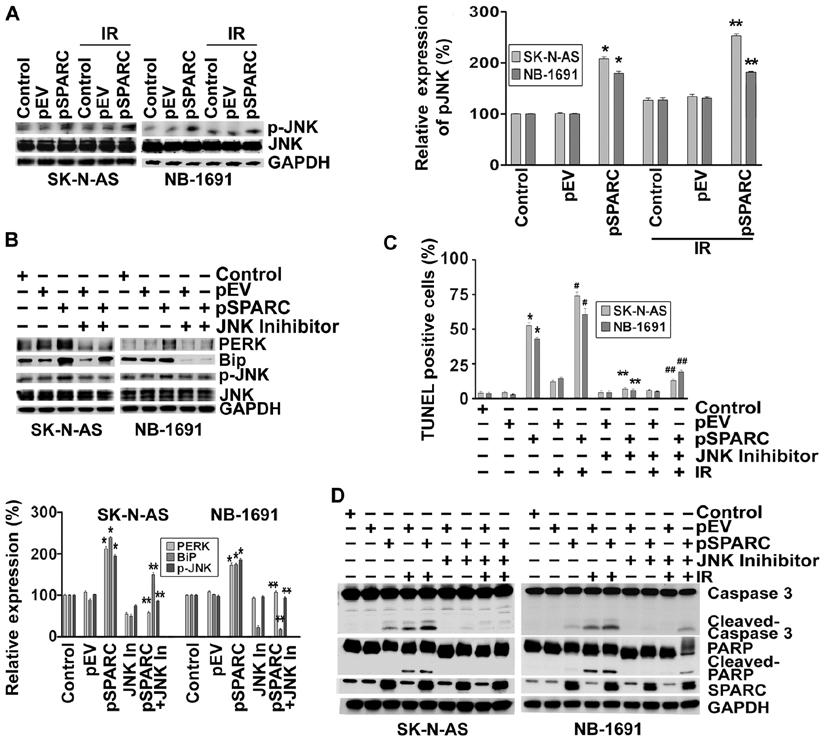
SPARC overexpression induces phosphorylation of JNK through endoplasmic reticulum (ER) stress. (A) SK-N-AS and NB-1691 neuroblastoma cells were seeded in dishes and left overnight. Cells were transfected with pEV or pSPARC and cultured. After 24 h, cells were irradiated with 8 Gy and incubated for another 16 h. Cells were collected and the cell lysates were subjected to western blotting for phospho-JNK (p-JNK) and JNK. GAPDH served as a loading control. Results are representative of three independent experiments. Phospho-JNK band intensities were quantified by densitometry using ImageJ (NIH) software and shown as the bar graph. Columns, mean of triplicate experiments; bars, ± SD. ^*^p<0.01 vs. pEV; ^**^p<0.01 vs. IR + pEV. (B) Neuroblastoma cells were transfected with pEV or pSPARC and cultured. After 24 h, cells were treated with JNK inhibitor for 16 h. Cells were collected and the lysates were subjected to western blotting for PERK, BiP, phospho-JNK (p-JNK) and JNK. GAPDH served as a loading control. Results are representative of three independent experiments. Densitometric analysis for PERK, BiP and p-JNK was performed using ImageJ (NIH) software and shown as the bar graph. Columns, mean of triplicate experiments; bars, ± SD. ^*^p<0.01 vs. pEV; ^**^p<0.01 vs. pSPARC. (C) Neuroblastoma cells were cultured in 8-well chamber slides and transfected and irradiated as described above. After irradiation, cells were treated with JNK inhibitor for 16 h. TUNEL assay was performed as described in Fig. 2B. The TUNEL-positive cell population was quantified and shown as the bar graph. Columns, mean of triplicate experiments; bars, ± SD. ^*^p<0.01 vs. pEV; ^**^p<0.01 vs. pSPARC; ^#^p<0.01 vs. IR + pEV; ^##^p<0.01 vs. IR + pSPARC. (D) Neuroblastoma cells were plated, transfected, irradiated and treated with JNK inhibitor as described above. Cells were collected and the cell lysates were subjected to western blotting for SPARC, caspase 3 and PARP. GAPDH served as a loading control. Results are representative of three independent experiments.

There is a wealth of knowledge pointing out the profound role of c-Jun N-terminal kinase (JNK) in apoptosis (23). In addition, it was shown that IRE1α can also initiate cell death through the activation of the JNK pathway (24). Hence, we sought to determine the effect of SPARC overexpression on phosphorylation of JNK. The results show that phospho-JNK levels increased ∼2-fold as a result of SPARC overexpression (Fig. 3A). To understand the involvement of JNK pathway and ER stress molecules in the apoptosis signaling cascade, SPARC-transfected neuroblastoma cells were treated with a JNK inhibitor after radiation. When phosphorylation of JNK was inhibited by the pharmacological inhibitor, the expression levels of ER stress molecules BiP and PERK were downregulated; these results illustrate that ER stress activation was initiated by the phosphorylation of JNK (Fig. 3B). It was also found that when activation of JNK was inhibited, the TUNEL positivity of pSPARC-transfected cells either alone or in combination with radiation was significantly diminished (Fig. 3C). Further, inhibition of JNK activity resulted in a marked decrease of cleavage of caspase 3 and PARP among pSPARC-transfected cells, thereby confirming the active involvement of JNK in regulating apoptosis in these cells (Fig. 3D).

### Endoplasmic reticulum stress regulates apoptosis

**Figure 4.**
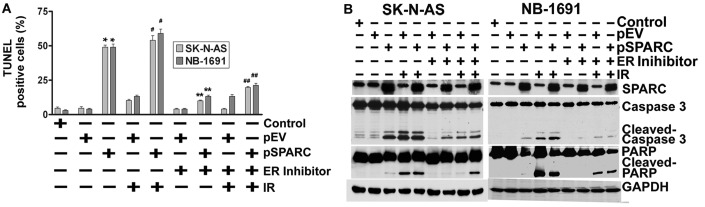
Endoplasmic reticulum (ER) stress regulates apoptosis. (A) SK-N-AS and NB-1691 neuroblastoma cells were seeded in 8-well chamber slides and left overnight. Cells were transfected with pEV or pSPARC and cultured. After 24 h, cells were irradiated with 8 Gy followed by an ER inhibitor and incubated for another 16 h. TUNEL assay was performed as described in Fig. 2B. The TUNEL-positive cell population was quantified and shown as the bar graph. Columns, mean of triplicate experiments; bars, ± SD. ^*^p<0.01 vs. pEV; ^**^p<0.01 vs. pSPARC; ^#^p<0.01 vs. IR + pEV; ^##^p<0.01 vs. IR + pSPARC. (B) Neuroblastoma cells were plated, transfected, irradiated and treated with ER inhibitor as described above. Cells were collected and the cell lysates were subjected to western blotting for SPARC, caspase 3 and PARP. GAPDH served as a loading control. Results are representative of three independent experiments.

Next, the contributory role of ER stress to induce apoptosis as a result of JNK activation by SPARC overexpression was tested using an ER stress inhibitor in pSPARC-transfected and irradiated neuroblastoma cells. As anticipated, inhibition of ER by a pharmacological inhibitor also significantly reduced the TUNEL positivity of SK-N-AS and NB-1691 neuroblastoma cells (Fig. 4A). Further, we also noted a sharp decrease in the activation of caspase 3 and cleavage of PARP among the pSPARC-transfected and ER inhibitor-treated cells (Fig. 4B).

### SPARC overexpression in combination with irradiation in an orthotopic neuroblastoma model suppresses tumor growth in vivo

**Figure 5.**
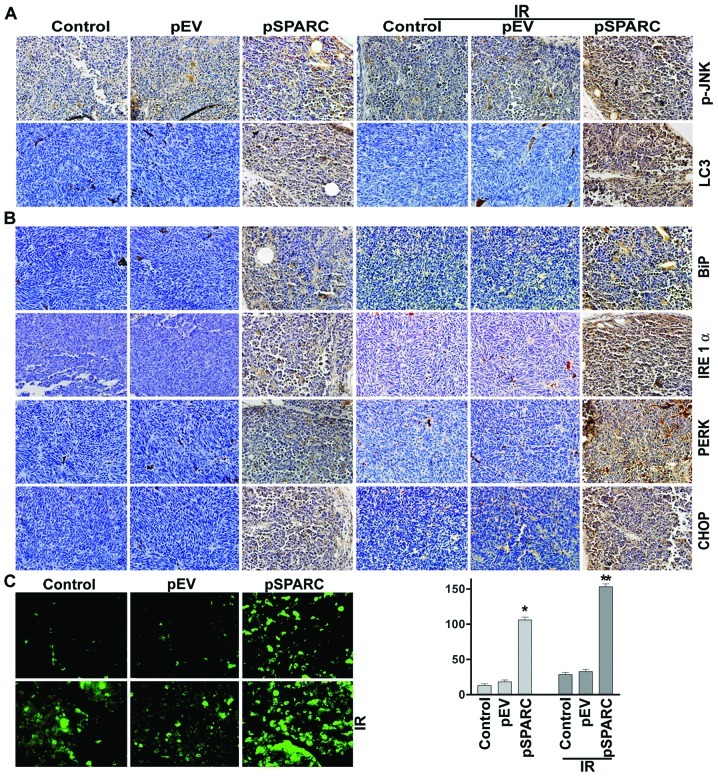
SPARC overexpression alone and in combination with radiation inhibits pre-established tumor growth *in vivo*. Neuroblastoma orthotopic tumor sections from mice injected with mock, pEV or pSPARC plasmids alone or in combination with radiation (IR) were analyzed as described in Materials and methods. (A) Immunohistochemical analysis for phospho-JNK (pJNK) and LC3 were carried out as described in Materials and methods. (B) Immunohistochemical analysis for BiP, IRE 1α, PERK and CHOP were carried out as described in Materials and methods. All results are representative of multiple tumors taken from five separate mice in each treatment group (magnification ×60). Inset, negative control. (C) Staining for TUNEL-positive cells in xenograft tissue sections from mock, pEV, and pSPARC alone or in combination with radiation (2 doses of 5 Gy)-treated mice was performed as described in Materials and methods. TUNEL-positive cells were quantified and shown as bar graph. Columns, mean of triplicate experiments; bars, ± SD. ^*^p<0.01 vs. pEV; ^**^p<0.01 vs. IR + pEV.

Based on the *in vitro* results, it could be proposed that SPARC overexpression invokes ER stress, which in turn enhances autophagy-mediated apoptosis in neuroblastoma. This hypothesis was tested by orthotopically implanting NB-1691 neuroblastoma cells in mice and treating with pSPARC alone and in combination with radiation. Increased SPARC expression levels were observed in pSPARC-treated tumors as compared to mock or pEV-treated tumors. The expression levels of phospho-JNK and LC3 were found to increase in tumors treated with pSPARC alone and in combination with radiation (Fig. 5A). Further, the ER stress molecules IRE 1α, BiP, PERK and CHOP were also expressed in elevated levels in pSPARC-treated tumors (Fig. 5B). TUNEL analysis confirmed pSPARC-induced apoptosis *in vivo* and a remarkable increase in apoptosis was observed with the combination treatment of pSPARC and radiation (Fig. 5C). The *in vivo* results thus corroborate the *in vitro* findings and support the hypothesis that ER stress plays a key role in regulating the induction of apoptosis in SPARC-overexpressed neuroblastoma cells. In addition, these results demonstrate the involvement of LC3 and active JNK as part of the signal pathway leading to apoptosis in the presence of SPARC overexpression and irradiation.

## Discussion

It is well established that the induction of apoptosis is critical to prevent the progression of any cancer. SPARC, through its inherent involvement in directing ECM deposition, cell-ECM interactions and growth factor signaling, plays numerous roles in regulating the multiple hallmarks of cancer including angiogenesis, migration, proliferation and survival (5). The results of the present study demonstrate that the combination treatment of SPARC overexpression and irradiation induces apoptosis in a synergistic manner in neuroblastoma cells. However, the tumor suppressive properties of SPARC are highly dependent on various aspects, especially cell phenotype and the tumor microenvironment (25,26). Hence, elucidation of the mechanisms underlying SPARC-mediated tumor suppression and the numerous confounding factors have been investigated in order to develop therapeutic strategies to confront cancer growth and metastasis. Activation of caspases is recognized as a critical event in most of the anticancer signaling pathways (27). SPARC overexpression alone or in combination with radiation treatment has been shown to enhance cleavage of caspase 3 and PARP in neuroblastoma cells. Further, our study demonstrates that SPARC overexpression directed ER stress as a function of apoptosis via activation of ER stress transducers (e.g., PERK, IRE 1α and BiP).

The ER performs diverse functions including protein folding and also plays a major role in calcium homeostasis (28). It is well known that protein folding occurs in the ER prior to transport to various extracellular surface or intracellular organelles. There is significant evidence suggesting the essential role of ER in the regulation of apoptosis as well as autophagy (16,29). Several types of cellular stress conditions can affect the protein folding process. Since unfolded or misfolded protein presents a threat to the cell, the ER lumen triggers the unfolded protein response (UPR) to circumvent cellular damage. The response to this ER stress induces activation of inositol-requiring endoplasmic reticulum-to-nucleus signal kinase (IRE)-1α (30). In addition, RNA-dependent protein kinase (PKR)-like ER kinase (PERK) is a ubiquitous short-term perturbation to ER stress that leads to transduction of luminal signals across the ER membrane to its cytosolic kinase domain (31). This, in turn, changes the reserve ER chaperone leading to expression of molecules like BiP. Given that BiP overexpression is known to suppress the UPR, enhanced expression of BiP contributes to effective ER stress response (32). Further, PERK amplification also leads to the phosphorylation of the α subunit of the translation factor, eIF2α, that ultimately inhibits protein synthesis by impeding the assembly of the 80s ribosome (30). This scenario presents pertinent evidence for the involvement of ER stress in the signaling cascade. We observed the upregulation of PERK, IRE 1α and BiP in both SK-N-AS and NB-1691 cells at an earlier time-point after SPARC transfection. These ER stress transducers in turn induced activation of the transcription factor CHOP (Fig. 2D).

Likewise, multiple pathways seem to be involved in ER stress-initiated apoptosis, including the formation of autophagosomes and activation of JNK as illustrated by the expression of autophagy markers and active participation of kinases in regulating apoptosis. The diverse signaling activities associated with SPARC overexpression also highlight the ER protein flux. Even though the entire mechanism associated with ER stress-mediated apoptosis is unclear, the downstream ER stress signaling could be correlated with activation of CHOP (33). Further, it has been previously shown that activated IRE recruits the scaffolding protein TRAF2 to the ER membrane, triggering the mitogen-activated protein (MAP) kinase cascade and leading to c-Jun N-terminal kinase (JNK) activation (22).

It is further apparent from the results that when active JNK was inhibited after SPARC overexpression, the ER stress transducers were downregulated; this result exemplifies the involvement of ER molecular chaperones in eliciting c-Jun N-terminal kinase as part of the signaling cascade. It was found that when phosphorylation of JNK was inhibited, apoptosis was inhibited in both cell lines, which confirms the active involvement of JNK in regulating apoptosis. In addition, when phosphorylation of JNK was inhibited, the expressions of ER stress molecules PERK, BiP and IRE were also downregulated, thereby demonstrating that ER stress activation induces phosphorylation of JNK. Further, the role of ER stress in directing apoptosis was tested using an ER stress inhibitor. We found that when ER stress was inhibited, apoptosis was significantly reduced (Fig. 4), which further confirms the role of ER stress in the signaling cascade associated with SPARC overexpression leading to autophagy-mediated apoptosis in neuroblastoma cells.

Autophagy involves sequestration of autophagosomes that eventually fuse with lysosomes leading to cellular degradation and has an important role in eukaryotic cells. Autophagy is regulated by a set of evolutionarily conserved autophagy-related (Atg) proteins. It was found that microtubule-associated protein light chain 3 II (LC3-II) is formed as a result of phosphoatidylethanolamine conjugation of LC3-I (also known as Atg8) indicate the formation of autophagosomes (34). Increased LC3 levels emphasize the coupling of UPR to autophagy in neuroblastoma. It has been previously demonstrated by the authors that SPARC overexpression leads to autophagy-mediated apoptosis in medulloblastoma (12). Even though the cross-talk between ER stress-induced autophagy and apoptosis is not completely understood, recent reports propose that PERK-eIF2α pathway or IRE-TRAF2-JNK pathway could be the crucial mediator of ER stress-induced autophagy (15).

This study also provides strong evidence that integrating radiation as part of the combination treatment intensified the degree of apoptosis by sensitizing the cells in a synergistic manner as indicated by flow cytometry analysis, TUNEL assay, and activation of apoptotic molecules like caspase 3 and cleavage of PARP. Given the complexity of events initiated as part of ER stress induction, it could be proposed that there is an interplay of multiple intracellular pathways during SPARC-induced autophagy and apoptosis in neuroblastoma cells. The efficacy of the proposed hypothesis when tested *in vivo* showed concomitant results in the expression of ER stress molecular chaperons as a function of increased SPARC levels. These results confirm that ER stress has a key role in regulating the induction of apoptosis in SPARC-overexpressed neuroblastoma cells. The immunohistochemical analysis of phospho-JNK and LC3 expression levels in response to increased SPARC levels and the combination treatment further corroborates the *in vitro* results. In conclusion the results impart new insights regarding ER stress-mediated apoptosis in SPARC-overexpressed cells that should be explored further as a potential therapeutic option for neuroblastoma.
